# What Research Has Been Conducted on Procrastination? Evidence From a Systematical Bibliometric Analysis

**DOI:** 10.3389/fpsyg.2022.809044

**Published:** 2022-02-02

**Authors:** Bo Yan, Xiaomin Zhang

**Affiliations:** School of Public Policy and Administration, Xi'an Jiaotong University, Xi'an, China

**Keywords:** procrastination, co-citation analysis, intellectual structure, CiteSpace, bibliometric analysis

## Abstract

Procrastination is generally perceived as a common behavioral tendency, and there are a growing number of literatures to discuss this complex phenomenon. To elucidate the overall perspective and keep abreast of emerging trends in procrastination research, this article presents a bibliometric analysis that investigates the panorama of overviews and intellectual structures of related research on procrastination. Using the Web of Science Database, we collected 1,635 articles published between 1990 and 2020 with a topic search on “procrastination” and created diverse research maps using CiteSpace and VOS viewer. Bibliometric analysis in our research consists of category distribution, keyword co-occurrence networks, main cluster analysis, betweenness centrality analysis, burst detection analysis, and structure variation analysis. We find that most research has focused on students' samples and has discussed the definition, classification, antecedents, consequences and interventions to procrastination, whereas procrastination in diverse contexts and groups remains to be investigated. Regarding the antecedents and consequences, research has mainly been about the relationship between procrastination and personality differences, such as the five-factor model, temperament, character, emotional intelligence, and impulsivity, but functions of external factors such as task characteristics and environmental conditions to procrastination have drawn scant attention. To identify the nature and characteristics of this behavior, randomized controlled trials are usually adopted in designing empirical research. However, the predominant use of self-reported data collection and for a certain point in time rather than longitudinal designs has limited the validation of some conclusions. Notably, there have been novel findings through burst detection analysis and structure variation analysis. Certain research themes have gained extraordinary attention in a short time period, have evolved progressively during the time span from 1990 to 2020, and involve the antecedents of procrastination in a temporal context, theoretical perspectives, research methods, and typical images of procrastinators. And emerging research themes that have been investigated include bedtime procrastination, failure of social media self-control, and clinical interventions. To our knowledge, this is almost the first time to conduct systematically bibliometric analysis on the topic of procrastination and findings can provide an in-depth view of the patterns and trends in procrastination research.

## Introduction

Procrastination is commonly conceptualized as an irrational tendency to delay required tasks or assignments despite the negative effects of this postponement on the individuals and organizations (Lay, 1986; Steel, 2007; Klingsieck, 2013). Poets have even written figuratively about procrastination, with such phrases as “*Procrastination is the Thief of Time*,” and “*Procrastination is the Art of Keeping Up with Yesterday*” (Ferrari et al., 1995). Literal meanings are retained today in terms of time management. The conceptualizations of procrastination imply inaction, or postponing, delaying, or putting off a decision, in keeping with the Latin origins of the term “pro-,” meaning “forward, forth, or in favor of,” and “-crastinus,” meaning “tomorrow” (Klein, 1971). Time delay is just the behavioral reflection, while personality traits, cognitive and motivational process, as well as contextual conditions are in-depth inducements to procrastination. Procrastination can be viewed as purposive and irrational delay so as to miss the deadlines (Akerlof, 1991; Schraw et al., 2007).

Procrastination is believed to be a self-regulation failure that is associated with a variety of personal and situational determinants (Hen and Goroshit, 2018). Specifically, research suggests that task characteristics (e.g., unclear instructions, the timing of rewards and punishment, as well as task aversiveness), personality facets (e.g., the five-factor model, motivation, and cognition), and environmental factors (e.g., temptation, incentives, and accountability) are the main determinants of procrastination (Harris and Sutton, 1983; Johnson and Bloom, 1995; Green et al., 2000; Wypych et al., 2018). Procrastination can be an impediment to success, and may influence the individual's mood, and increase the person's anxiety, depression, and low self-esteem (Ferrari, 1991; Duru and Balkis, 2017). Furthermore, a person with procrastination is prone to poor performance, with lower exam scores, slower job promotions, and poorer health (Sirois, 2004; Legood et al., 2018; Bolden and Fillauer, 2020). Importantly, if policymakers postpone conducting their decision-making until after the proper timing, that procrastination can cause a significant and negative impact on the whole society, such as the cases with the COVID-19 pandemic management in some countries (Miraj, 2020).

In practice, procrastination is stable and complex across situations, ranging from students' academic procrastination, to staffs' work procrastination, to individuals' bedtime procrastination, to administrative behavior procrastination when government organizations face multiple tasks in national governance, and even to delayed leadership decision-making in crisis situations in global governance (Nevill, 2009; Hubner, 2012; Broadbent and Poon, 2015; Legood et al., 2018). As for science research, procrastination has attracted more and more attention and been studied extensively. Personally, possible explanations for emerging research focuses mainly consist of two aspects. On one hand, procrastination with high prevalence and obvious consequences highlights the importance to explore the complex phenomenon deeply, especially the meteoric rise in availability of information and communications technologies (ICTs) amplifies chronic procrastination, such as problematic social media use, smartphone addictions as well as mobile checking habit intrusion (Ferrari et al., 2007; Przepiorka et al., 2021; Aalbers et al., 2022). On the other hand, more and more basic and milestone research emerges in large numbers, which set the foundation for latecomer' further exploration toward procrastination. In particular, it can't be ignored the efforts of those productive authors in different periods to drive the knowledge development of procrastination.

Procrastination research has experienced tremendous expansion and diversification, but systematic and overview discussion is lacking. Several meta-analyses about procrastination have emerged, but they emphasize more on specific topics (Steel, 2007; Sirois et al., 2017; Malouff and Schutte, 2019). Furthermore, the number of newly published articles is increasing, so it becomes difficult to fully track the relevant domain literature. In order to grasp knowledge development about the fast-moving and complex research field, bibliometric analysis is necessary to construct diagram-based science mapping, so as to provide a comprehensive and intuitive reference for subsequent researchers. Thus, this article emphasizes on the following major research question: what is the intellectual base and structure of procrastination research? How does the emerging direction of procrastination develop? In our research, bibliometric analysis included the annual distribution of literature, distribution of categories, keyword co-occurrence networks, main research clusters, high citation betweenness centrality, and the strongest citation bursts, as well as the recent publications with transformative potential, in order to look back on the early development of procrastination research and look forward to the future transformation of that research. For both scholars and members of the public, this study can comprehensively enhance their understanding of procrastination and can provide overall perspectives for future research.

## Data and Methodology

Bibliometric analysis is a quantitative method to investigate intellectual structures of topical field. On the basis of co-citation assumption that if two articles are usually cited together, then there are high associations between those articles, bibliometric analysis can reflect the scientific communicational structures holistically (Garfield, 1979; Chen et al., 2012). Bibliometric techniques, such as CiteSpace, VOSviewer, HistCite, can generate the science maps based on plenty of literature concerning certain domain. Through the process of charting, mining, analyzing, sorting, and displaying knowledge, science mapping can extract pivotal information from huge complex literature, present knowledge base and intellectual structure of a given field visually, then researchers even general individual can quickly grasp one subject's core structure, development process, frontier field and the whole knowledge framework (Chen, 2017; Widziewicz-Rzonca and Tytla, 2020). Bibliometric analysis is commonly regarded as a complementary method to traditional structured literature reviews such as narrative analysis and meta-analysis (Fang et al., 2018; Jiang et al., 2019). Traditional literature analysis tends to labor intensive with subjective preferences, and faces difficulties in analyzing larger body of literature, whereas bibliometric analysis provides a more objective approach for investigating considerable literature's intellectual structure through statistical analysis and interactive visual exploration.

In order to master the characteristics of procrastination research, the study adopted the bibliometric software of CiteSpace and VOSviewer to analyze the literature on procrastination during the time period 1990–2020. The software tool VOSviewer is designed for creating maps of authors, journals, and keyword co-occurrences based on network data (van Eck and Waltman, 2010), whereas CiteSpace is applied to conduct co-citation analysis, including centrality betweenness analysis, burst detection, and the emerging trends of research (Chen, 2006, 2017). In our study, we adopted the CiteSpace (5.7.R1) and VOSviewer (1.6.15) software together. Specifically, co-citation analysis mainly depends on CiteSpace software, and co-occurrence analysis is conducted through VOS viewer (Markscheffel and Schroeter, 2021).

Though there is one similar bibliometrics analysis toward this topic (Tao et al., 2021), related research just focuses on academic procrastination, and mainly conducts co-occurrence analysis using VOSviewer, so as to there is a lack of analysis to core co-citation structures including high betweenness centrality articles, citation burst research and structure variation analysis. To offer insight into the intellectual structure of procrastination research, we further employ CiteSpace — a java application including bibliometric analysis, data mining algorithms and visualization methods developed by Chen — to visualize and elucidate vital trends and pivotal points about knowledge development.

To conduct our bibliometric analysis of procrastination research, we collected bibliographic records from the Web of Science Core Collection as of December 31, 2020. Web of Science is currently the most relevant scientific platform regarding systematic review needs, allowing for a “Topic” query, including searching a topic in the documents' “title”, “abstract”, “author keywords” and “keywords plus” of the documents being reviewed (Yi et al., 2020). A topic search strategy is broad enough to be used in science mapping (Olmeda-Gomez et al., 2019). Given the aim of the study, records were downloaded if they had the term “procrastination” in the “Topic” field. After restricting the type of publication to “Article” for the years 1900–2020, we had searched 2105 papers about procrastination research.

**Figure 1:**
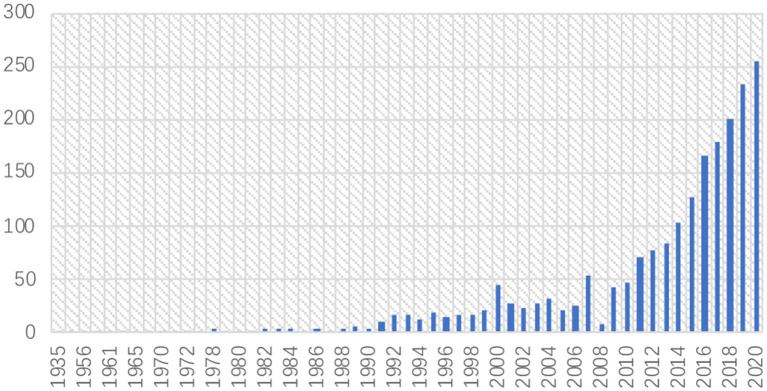
Distribution of publications on the topic of procrastination, 1900-2020.

Figure 1 shows the yearly distribution of 2105 literature during 1900–2020, and it can be classified into three phases. In phase I (1900–1989), the annual number of publications never exceeded 10. In phase II (1990–2010), the annual quantity gradually increased from 11 papers in 1991 to 48 in 2010. The annual number of publications had begun to grow in this period, but remained below 50 papers yearly. In phase III (2011–2020), however, the procrastination research experienced a dramatic growth, with 255 literature in the year 2020. Although procrastination research appeared as early as 1900s, it had a stable total volume until the 1990s, when it developed sustained growth, and that growth became extraordinary during the 2010s. Therefore, this research emphasized centered on 1,635 literature that were published during the time span 1990–2020.

## Panoramic Overview of Procrastination Research

### Category Distribution

**Figure 2:**
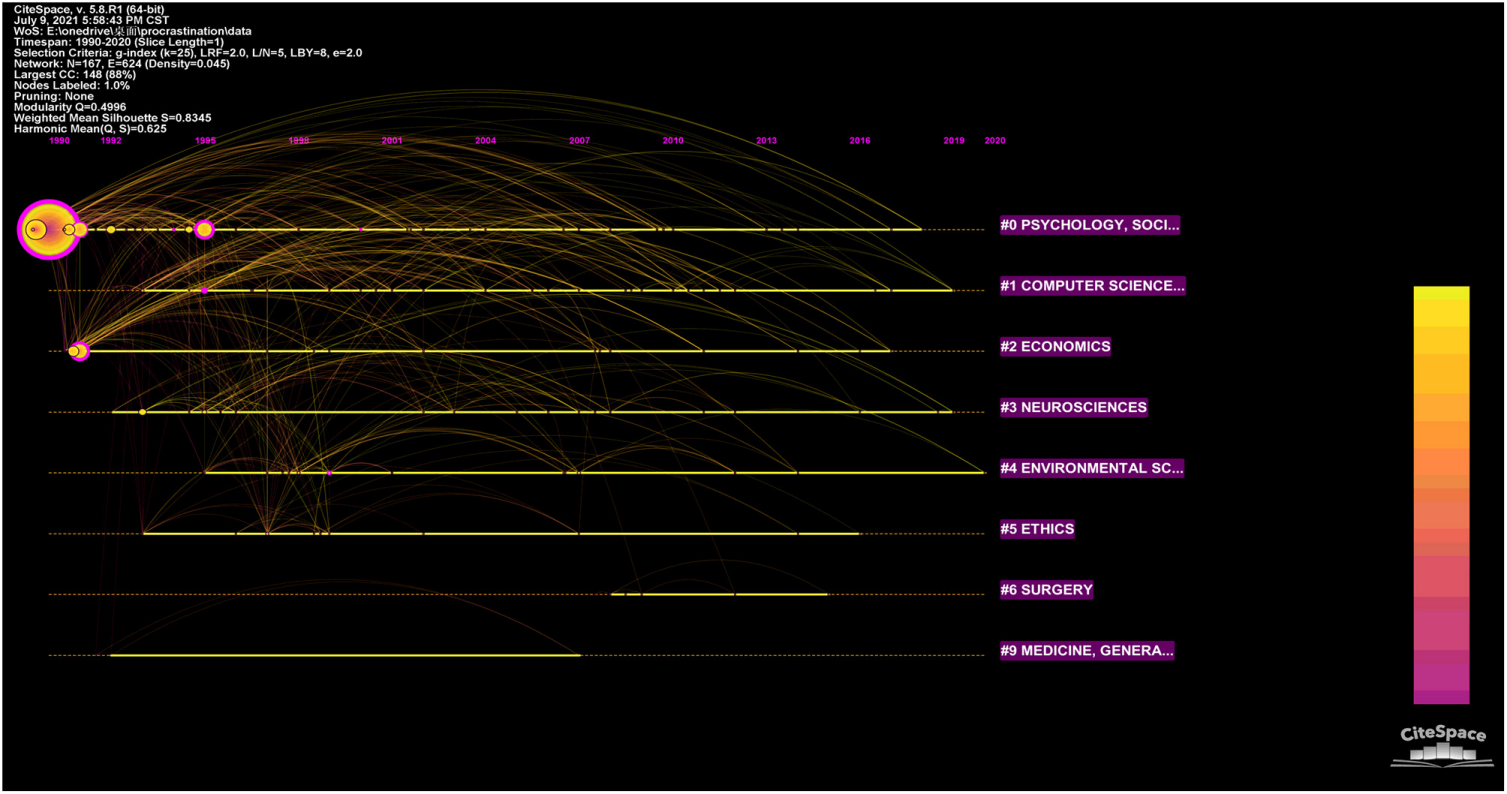
Distribution of categories involved in procrastination research.

Procrastination research has been attracting increasing attention from scholars, and it has been successfully integrated into various scientific fields. With the help of CiteSpace software, we present in Figure 2 the timelines of the various disciplines that are involved in procrastination research, and the cumulative numbers of literature that have been published.

As Figure 2 shows, the size of node on the horizontal lines represents the quantity of literature published. Node colors denote the range of years of occurrence, and purple outlining is an indication of those articles with prominent betweenness centrality, and red nodes present references with high citation burst (Chen, 2017). Besides, the uppermost line shows the timeline of different disciplines, and the numbers on the longitudinal lines describe the distinct categories of procrastination research, of which are arranged vertically in the descending order of cluster's size. Clusters are numbered from 0, i.e Cluster #0 is the largest cluster and Cluster #1 is the second largest one. Specifically, the earlier research about procrastination occurs in the Psychology and Social Science disciplines. Subsequently, research has expanded into Computer Science and Information Systems, Economics, the Neurosciences, the Environmental Sciences, Ethics, Surgery, and general Medicine. As the connections arc in the Figure 2 presents, those categories #0 Psychology and Social Sciences, #1 Computer Science, and #2 Economics interact actively, but the interdisciplinary research about the remaining categories, such as #9 Medicine, #5 Ethics, and #4 Environmental Science, is not active.

Our analysis of the category distribution reveals two aspects of the characteristics about procrastination research. One, related research mostly has its roots in the Psychology and Social Science disciplines, and interdisciplinary research needs to be improved. And Two, the foundational literature dates back to the 1990s, and transformational exploration is currently needed in order to further develop the research on procrastination.

### Keyword Co-occurrence Network: Core Contents

**Figure 3:**
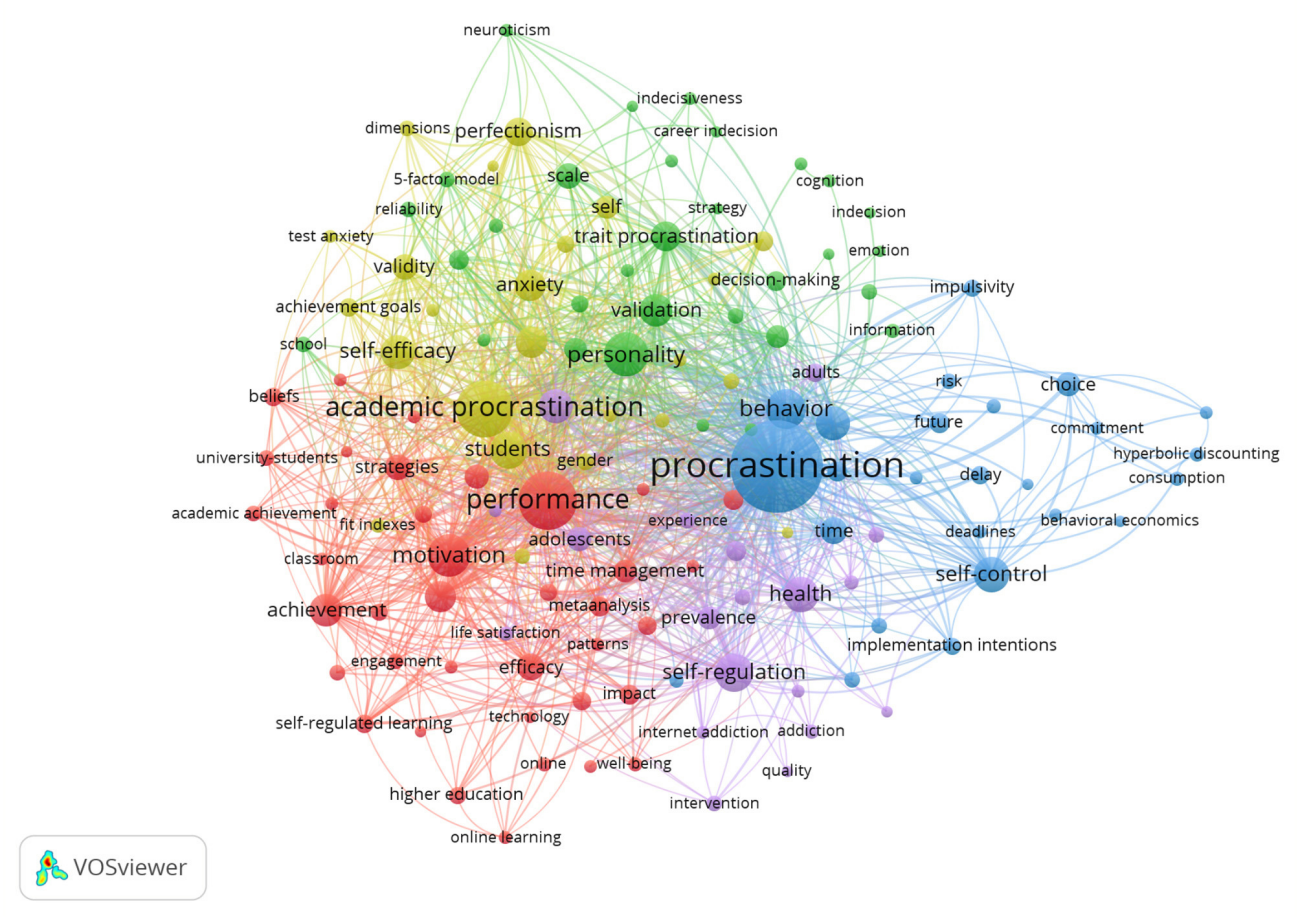
Keywords co-occurrence network for procrastination research.

Analysis of co-occurring keywords is often used to obtain the content of research fields. Using the VOS viewer, we obtained a total of 5,203 keywords and created a co-occurrence network. As mentioned above, the size of a node represents the number of times that a specific keyword occurs. Several keywords turn up frequently, such as Procrastination, Performance, Academic Procrastination, Motivation, Personality, Self-regulation, Self-control, and Behavior. To create a readable map, the “minimum number of occurrences” is set to 20, and the final network includes 90 high-frequency keywords and five clusters with 2,650 links, as is shown in Figure 3.

Among the five clusters depicted in Figure 3, the blue cluster is mainly related to the definition of procrastination, with keywords such as Procrastination, Delay, Deadlines, Choice, Self-Control, and Implementation Intentions. Procrastination is a complex phenomenon, and previous research has elaborated on the core traits about procrastination from various dimensions. Mainstream views hold that procrastination can be defined as the intentional delay of work because of a self-regulation failure, time-management inefficiency, short-term benefits, a gap between intention and action (Tice and Baumeister, 1997; Steel, 2007; Pychyl and Flett, 2012; Klingsieck, 2013), or missing a deadline and causing negative outcomes (Johnson and Bloom, 1995; Howell and Watson, 2007; Sirois, 2021).

The cluster in red in Figure 3 involves procrastination performance in relation to different life-domains, including Academic Achievement, Life Satisfaction, Online Learning, and Technology Uses. Previous research has elaborated on procrastination as being negatively correlated with performance. However, intrinsic motivation, self-regulated learning, and time-management have been shown to relieve the procrastination behavior (Wolters, 2003; Howell and Watson, 2007; Baker et al., 2019).

The green cluster highlights traits associated with procrastination. Related research in that cluster mostly discusses the correlation between the five-factor model (neuroticism, extraversion, openness to experience, agreeableness, conscientiousness) and procrastination (Schouwenburg and Lay, 1995). In addition, personality traits including indecisiveness, indecision, and perfectionism have been elaborated upon (Klingsieck, 2013; Tibbett and Ferrari, 2019). Furthermore, to measure the trait of procrastination itself, various scales have been developed, such as the General Procrastination Scale, Decisional Procrastination Questionnaire, Procrastination at Work Scale, Irrational Procrastination Scale, Adult Inventory of Procrastination Scale and so on (Lay, 1986; Ferrari et al., 1995; Steel, 2010; Metin et al., 2016). The validity and reliability of those scales have also been investigated fully.

The cluster presented in yellow depicts studies that focuses on academic procrastination, and especially those that discuss the antecedents of the prevalent behavior, such as Anxiety, Perfectionism, Self-efficacy, Depression, and Stress (Schraw et al., 2007; Goroshit, 2018). Owing to their accessibility for use as a research sample, a large body of procrastination research has chosen students in an academic setting as the research objects. Researchers have found that academic procrastination is an impediment to academic performance, especially for very young students. Notably, too, female students may perform lower levels of academic procrastination than males do.

The last cluster, presented in purple, relates to chronic procrastination's involvement in health and addiction, for either adults or adolescents. Discussion about chronic procrastination is growing, and interventions can be effective in relieving this behavior.

From the analysis of co-occurrence keywords, we can infer that procrastination research has been developing steadily. The fundamental discussion has become more adequate and persuasive in regard to the definition, the individual differences, and the antecedents of procrastination, and a discussion of how to relieve the behavior has begun.

### Main Research Cluster: Core Theme and Hot Topics

Comparing to keyword co-occurrence network analyses, cluster analysis can help us grasp the primary themes in procrastination research. Clusters are based on the assumption that if two references are often cited together, they may be associated in some way (Chen et al., 2012; Pan et al., 2019). Eventually, related references shape diverse co-citation networks. Clustering is a procedure to classify co-cited references into groups, with references in the same clusters being tightly connected with each other but loosely associated with other clusters (Chen et al., 2010).

Based on the references of the top 50 articles with the most citations every year (if the number was less than 50 in a certain year, then all of the articles were combined), the final network contained 982 references and we were able to develop the final cluster landscape. Two procedures are used to label each cluster: (1) retrieval of keywords from the citing articles using the log likelihood ratio, and (2) retrieval of terms contained in the cited articles with latent semantic indexing (Olmeda-Gomez et al., 2019). In our research, we adopted the log-likelihood ratio (LLR) method to label the clusters automatically. Given the related structural and time-based values, articles in the co-citation network are assigned to each cluster. Eventually, the network was divided into 23 co-citation clusters.

**Figure 4:**
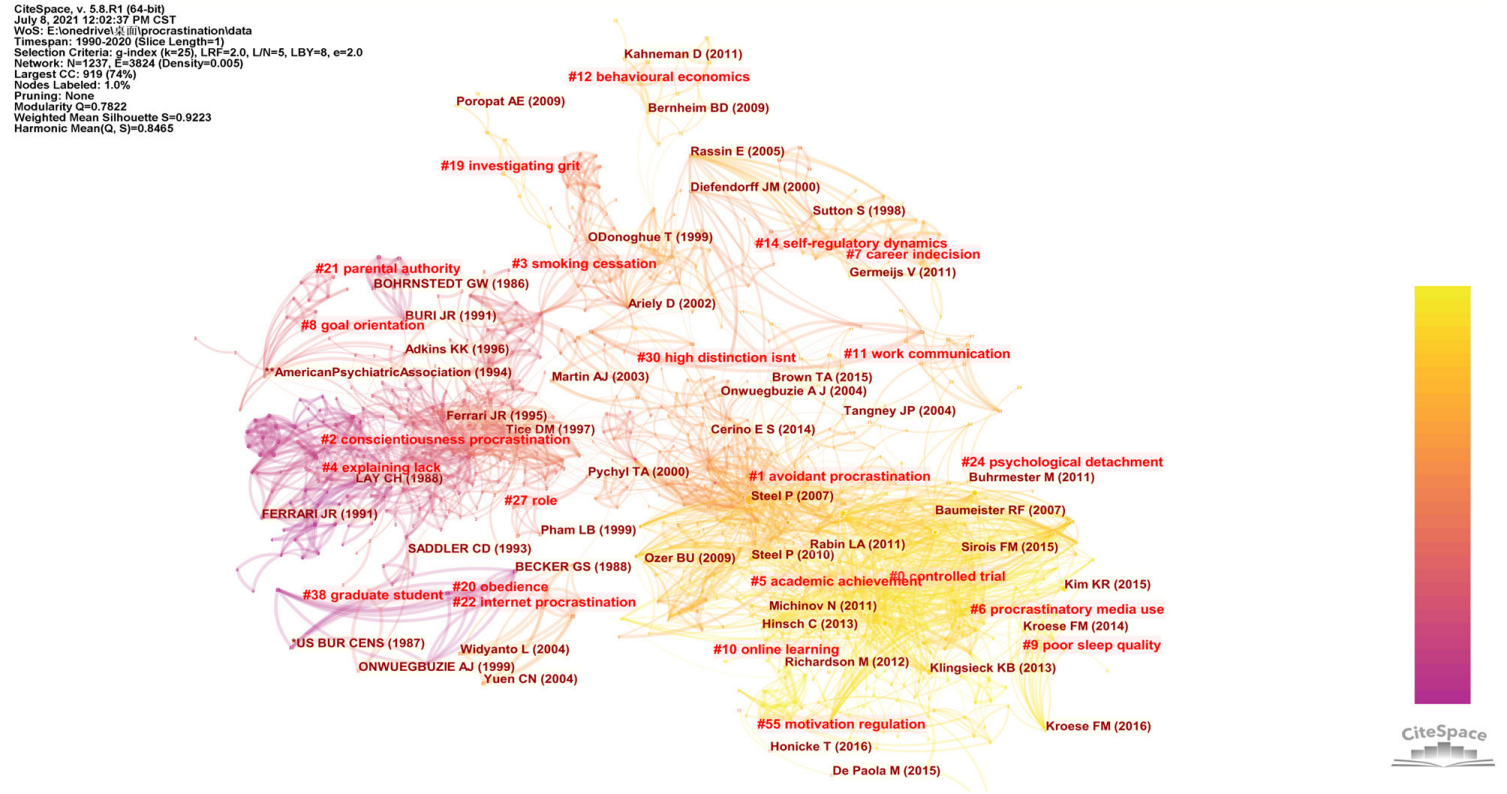
Landscape view of co-citation network of procrastination research.

In addition, two critical parameters, silhouette and modularity, are used to measure whether clusters are available and whether they are well-constructed. Silhouette indicates the homogeneity of clusters, whereas modularity measures whether the network is reasonably divided into independent clusters. The silhouette value ranges from −1 to 1, and the modularity score ranges from 0 to 1. When values of the two metrics are high, the co-citation network is well-constructed (Chen et al., 2010; Widziewicz-Rzonca and Tytla, 2020). As is shown in Figure 4, the mean silhouette score of 0.9223 suggested that the homogeneity of these clusters was acceptable, and the modularity score of 0.7822 indicated that the network was reasonably divided.

In our research, we summed the largest nine clusters. As is shown in Table 1, the silhouette value for all clusters was higher than 0.8, suggesting the references in each cluster were highly homogeneous. The labels of these clusters were controlled trial, avoidant procrastination, conscientiousness procrastination, smoking cessation, explaining lack, academic achievement, procrastinatory media use, career indecision, and goal orientation.

**Table 1:** Summary of the nine largest clusters in procrastination research.

**Cluster ID**	**Size**	**Silhouette**	**Label (LLR)**	**Mean (Year Cited)**
0	182	0.855	Controlled trial	2014
1	148	0.836	Avoidant procrastination	2005
2	144	0.938	Conscientiousness procrastination	1994
3	72	0.989	Smoking cessation	2000
4	65	0.97	Explaining lack	1988
5	58	0.903	Academic achievement	2009
6	33	0.988	Procrastinatory media use	2013
7	31	0.99	Career indecision	2006
8	28	0.981	Goal orientation	1995

In Table 1, the year in the far-right column indicated the average year when the reference was cited. Ranking the clusters by the mean cited year, we can follow the development of research themes. During the 1990s, research themes focused on discussions about the antecedents of procrastination. For example, Lay (1988) discussed that the self-regulation model cannot explain procrastination fully, and errors in estimations of the time taken to complete a task may be attributed to procrastination. Procrastinators were thought to tend to lack conscientiousness and goal orientation as well as to be motivated by neurotic avoidance (Ferrari et al., 1995; Elliot and Harackiewicz, 1996). Besides, procrastination was prevalent throughout our lifespan, and empirical research on procrastination conducted through controlled trials had considered various settings or scenarios, such as academic procrastination, smoking cessation, career indecision, and in the most recent years, media use (Klassen et al., 2008; Germeijs and Verschueren, 2011; Du et al., 2019). Because procrastination was negatively associated with performance, life satisfaction, health and well-being, research on procrastination avoidance and intervention, including strengths-based training and cognitive behavioral therapy had attracted the most attention from scholars (van Eerde, 2003; Balkis and Duru, 2016; Visser et al., 2017).

## Intellectual Structure of Procrastination Research

Co-citation analysis and clustering analysis form the cornerstone for bibliometric investigation (Olmeda-Gomez et al., 2019), especially for the microscopic intellectual structures of the science, such as betweenness centrality, burst detection, and structural variation analysis (Pan et al., 2019). Based on the cited references network during the period of 1990–2020, we generated a landscape visualization of intellectual structures about procrastination research. The section consists of three parts: (1) Betweenness Centrality Analysis captures the bridge nodes, which represents the landmark and pivotal literature of a scientific field (Freeman, 1978). (2) Burst Detection Analysis is used to detect the emergent and sharp increases of interest in a research field (Kleinberg, 2003), which is a useful method for easily tracing the development of research focus and research fronts. (3) Structural Variation Analysis (SVA) is an optional measurement to identify whether newly published articles have the potential to transform the citation network in the latest years. Newly published articles initially have fewer citations and may be overlooked. To overcome the limitation, structural variation analysis often employs zero-inflated negative binomial (ZINB) and negative binomial (NB) models to detect these transformative and potential literature (Chen, 2013).

### Betweenness Centrality Analysis

Literature with high betweenness centrality tends to represent groundbreaking and landmark research. On the basis of our co-citation network on procrastination research for the period 1990–2020, we chose the top 10 articles to explore (see Supplementary Material for details). Related research mainly focuses on three areas.

### Definition and Classification of Procrastination

Procrastination is described as the postponement of completion of a task or the failure to meet deadlines, even though the individual would meet adverse outcomes and feel uncomfortable as a result (Johnson and Bloom, 1995). Extracting from authoritative procrastination scales, Diaz-Morales et al. (2006) proposed a four-factor model of procrastination: dilatory behaviors, indecision, lack of punctuality, and lack of planning. Procrastination is commonly considered to be a pattern of self-regulation failure or self-defeating behavior (Tice and Baumeister, 1997; Sirois and Pychyl, 2013).

The most popular classification is the trinity of procrastination: decisional, arousal, and avoidant procrastination (Ferrari, 1992). Using the General Behavioral Procrastination Scale and Adult Inventory of Procrastination Scale, Ferrari et al. (2007) measured the difference between arousal and avoidant procrastination, and they elaborated that those two patterns of procrastination showed similarity and commonality across cultural values and norms. However, by conducting a meta-analytic review and factor analyses, Steel (2010) found that evidence for supporting the tripartite model of procrastination may not be sufficient. Research has reached a consensus about the basic definition of procrastination, but how to classify procrastination needs further discussion.

### Procrastination Behavior in a Temporal Context

Procrastination is related to time management in its influence on one's behavior. Non-procrastinators or active procrastinators have better time control and purposive use of time (Corkin et al., 2011). However, time management is an obstacle to procrastinators. From the temporal disjunction between present and future selves, Sirois and Pychyl (2013) pointed out that procrastinators tended to give priority to short-term mood repair in the present, even though their future self would pay for the inaction. Similarly, in a longitudinal study Tice and Baumeister (1997) pointed out that maladjustment about benefits-costs in participants' timeframe shaped their procrastination. When a deadline is far off, procrastination can bring short-term benefits, such as less stress suffering and better health, whereas early benefits are often outweighed by possible long-term costs, including poor performance, low self-esteem, and anxiety. These viewpoints confirm that procrastination is a form of self-regulation failure, and that it involves the regulation of mood and emotion, as well as benefit-cost tradeoffs.

### Causes of and Interventions for Procrastination

Procrastination shows significant stability among persons across time and situations. Predictors of procrastination include personality traits, task characteristics, external environments, and demographics (Steel, 2007). However, typically, empirical research has mostly focused on the relationship between the five-factor model and procrastination behavior. Johnson and Bloom (1995) systematically discussed five factors of personality to variance in academic procrastination. Research also had found that facets of conscientiousness and neuroticism were factors that explained most procrastination. In alignment with these findings above, Schouwenburg and Lay (1995) elaborated that procrastination was largely related to a lack of conscientiousness, which was associated with six facets: competence, order, dutifulness, achievement-striving, self-discipline, and deliberation. Meanwhile, impulsiveness (a facet of neuroticism) has some association with procrastination, owing to genetic influences (Gustavson et al., 2014). These discussions have established a basis for research about personality traits and procrastination (Flett et al., 2012; Kim et al., 2017).

To relieve procrastination, time management (TM) strategies and clinical methods are applied in practice. Glick and Orsillo (2015) compared the effectiveness of those interventions and found that acceptance-based behavior therapies (ABBTs) were more effective for chronic procrastinators. Regarding academic procrastination, Balkis (2013) discussed the role of rational beliefs in mediating procrastination, life satisfaction, and performance. However, there is no “Gold Standard” intervention for procrastination. How to manage this complex behavior needs further investigation.

### Burst Detection Analysis

**Figure 5:**
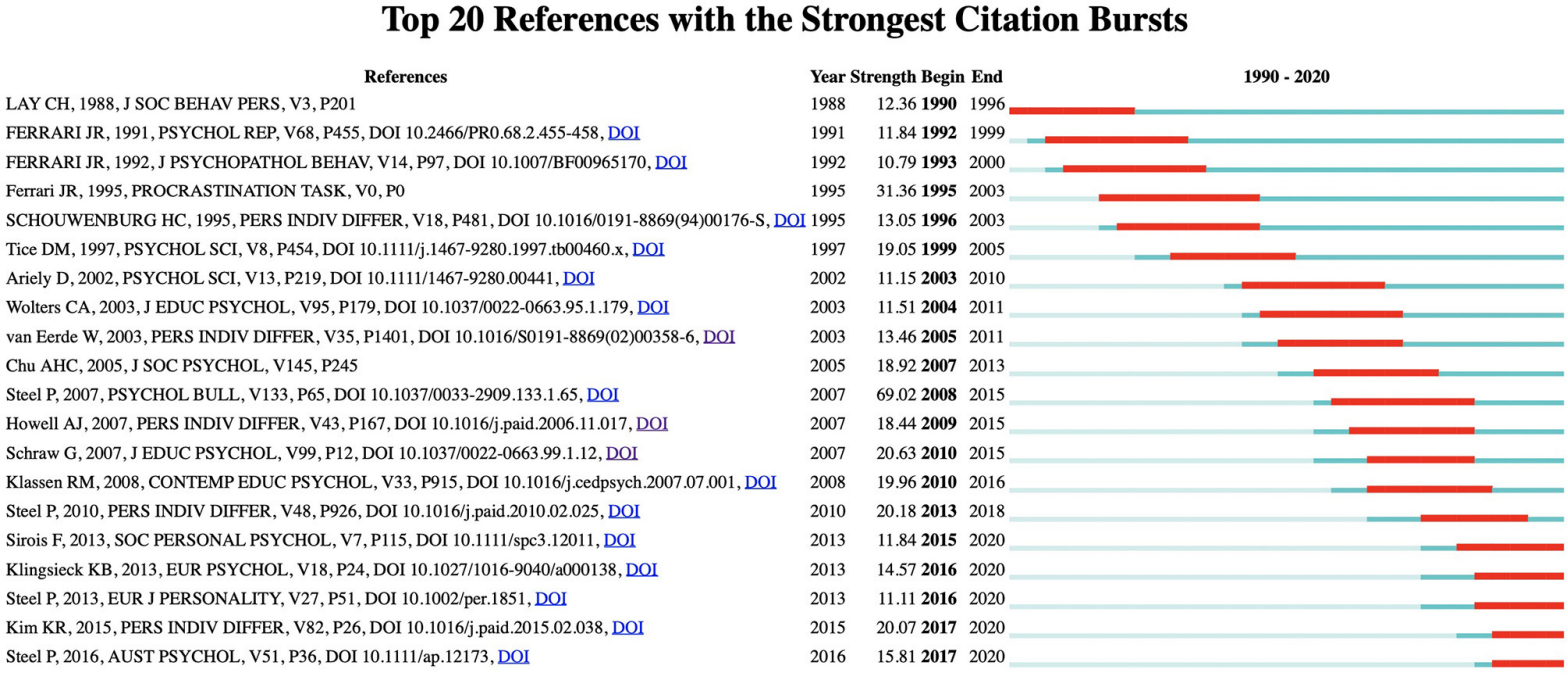
Top 20 references with the strongest citation bursts.

A citation burst indicates that one reference has gained extraordinary attention from the scientific community in a short period of time, and thus it can help us to detect and identify emergent research in a specialty (Kleinberg, 2003). A citation burst contains two dimensions: the burst strength and the burst status duration. Articles with high strength values can be considered to be especially relevant to the research theme (Widziewicz-Rzonca and Tytla, 2020). Burst status duration is labeled by the red segment lines in Figure 5, which presents active citations' beginning year and ending year during the period 1990-2020. As can be seen in Figure 5, we ranked the top 20 references (see Supplementary Material for details) with the strongest citation bursts, from the oldest to the most recent.

To systematically investigate the active areas of procrastination research in different time periods, we divided the study's overall timespan into three time periods. During the period 1990 through 1999, there were six references with high citation bursts, with two of them by Ferrari and a third by Ferrari, Johnson, and McCown. Subsequently, in 2000 through 2009, there were eight reference bursts, and the meta-analysis and theoretical review by Steel (2007) had the highest citation burst among those 20 references. From the period 2010 through 2020, six references showed high citation bursts.

#### Period I (1990–1999): Preliminary Understanding of Procrastination's Antecedents

How one defines procrastination is important to interventions. During the early period of procrastination research, scholars paid significant attention to define procrastination and discuss its antecedents. Time delay in completing tasks constitutes the vital dimension that distinguishes procrastination behavior, and that distinction has set the foundation for future exploration of the behavior. Lay (1988) found that errors in estimations of time led to procrastination, then identified two types of procrastinators: pessimistic procrastinators and optimistic ones, according to whether one is optimistic or pessimistic about judgments of time. In addition, the timeframe or constraint scenario influences one's behavioral choices. Procrastinators tend to weigh short-term benefits over long-term costs (Tice and Baumeister, 1997).

However, time delay is just a behavioral representation, and personality traits may be in-depth inducements to procrastination behavior (Ferrari, 1991; Ferrari et al., 1995). Schouwenburg and Lay (1995) empirically studied and elaborated upon the relationship between the five-factor model and procrastination facing a sample of students, and their findings showed consistency with research by Ferrari (1991) which demonstrated that the trait facets of lacking conscientiousness and of neurotic avoidance were associated with procrastination. In addition, Ferrari (1992) evaluated two popular scales to measure procrastination: the General Procrastination (GP) scale and the Adult Inventory for Procrastination (AIP) scale. Regarding the measurement of procrastination, a variety of scales have been constructed to further enhance the development of procrastination research.

#### Period II (2000–2009): Investigation of Cognitive and Motivational Facets and Emergence of Various Research Methods

During period II, procrastination research with high citation bursts focused largely on two dimensions: behavioral antecedences and empirical methods. On one hand, discussions about cognitive and motivational antecedents spring up. A series of studies find that cognitive and motivational beliefs, including goal orientation, perceived self-efficacy, self-handicapping, and self-regulated learning strategies, are strongly related to procrastination (Wolters, 2003; Howell and Watson, 2007; Klassen et al., 2008). Specifically, Howell and Watson (2007) examined the achievement goal framework with two variables, achievement goal orientation and learning strategies usage, in which four types of goal orientation can be derived by the performance vs. mastery dimension and the approach vs. avoidance dimension. Their research found that procrastination was attributed to a mastery-avoidance orientation, whereas it was adversely related to a mastery-approach orientation. Moreover, Chu and Choi (2005) identified two types of procrastinators, active procrastinators versus passive procrastinators, in terms of the individual's time usage and perception, self-efficacy beliefs, motivational orientation, stress-coping strategies, and final outcomes. This classification of procrastinators has aroused a hot discussion about procrastination research (Zohar et al., 2019; Perdomo and Feliciano-Garcia, 2020). Cognitive and motivational antecedents are complementary to personality traits, and the antecedents and traits together reveal the complex phenomenon.

In addition, there are various research methods being applied in the research, such as meta-analyses and grounded theory. Having the strongest citation burst in period II, research that was based on a meta-analysis of procrastination by Steel (2007) elaborated on temporal motivation theory (TMT). Temporal motivational theory provides an innovative foothold for understanding self-regulation failure, using four critical indicators: expectancy, value, sensitivity to delay, and delay itself. Similarly, van Eerde (2003) conducted a meta-analysis to examine the relationship between procrastination and personality traits, and proposed that procrastination was negatively related to conscientiousness and self-efficacy, but was also actively associated with self-handicapping. Procrastinators commonly set deadlines, but research has found that external deadlines may be more effective than self-imposed ones (Ariely and Wertenbroch, 2002). Furthermore, Schraw et al. (2007) constructed a paradigm model through grounded theory to analyze the phenomenon of academic procrastination, looking at context and situational conditions, antecedents, phenomena, coping strategies, and consequences. These diverse research methods are enhancing our comprehensive and systematical understanding of procrastination.

#### Period III (2010–2020): Diverse Focuses on Procrastination Research

After nearly two decades of progressive developments, procrastination research has entered a steady track with diverse current bursts, on topics such as type distinction, theoretical perspective, temporal context, and the typical image of procrastinators. Steel (2010) revisited the trinity of procrastination — arousal procrastinators, avoidant procrastinators, and decisional procrastinators — and using the Pure Procrastination Scale (PPS) and the Irrational Procrastination Scale (IPS), he found that there was no distinct difference among the three types. Regarding research settings, a body of literature has focused on academic procrastination in-depth, and that literature has experienced a significant citation burst (Kim and Seo, 2015; Steel and Klingsieck, 2016). For example, academic procrastination is associated more highly with performance for secondary school students than for other age groups.

Notably, theoretical discussions and empirical research have been advancing synchronously. Klingsieck (2013) investigated systematic characteristics of procrastination research and concluded that theoretical perspectives to explain the phenomenon, whereas Steel and Ferrari (2013) portrayed the “typical procrastinator” using the variables of sex, age, marital status, education, community location, and nationality. Looking beyond the use of time control or time perception to define procrastination, Sirois and Pychyl (2013) compared the current self and the future self, then proposed that procrastination results from short-term mood repair and emotion regulation with the consequences being borne by the future self. In line with the part of introduction, in the last 10 years, research on procrastination has flourished and knowledge about this complex phenomenon has been emerging and expanding.

### Structure Variation Analysis

Structure variation analysis (SVA) can predict the literature that will have potential transformative power in the future. Proposed by Chen (2012), structure variation analysis includes three primary metrics — the modularity change rate, cluster linkage, and centrality divergence — to monitor and discern the potential of newly published articles in specific domains. The modularity change rate measures the changes in and interconnectivity of the overall structure when newly published articles are introduced into the intellectual network. Cluster linkage focuses on these differences in linkages before and after a new between-cluster link is added by an article, whereas centrality divergence measures the structural variations in the divergence of betweenness centrality that a newly published article causes (Chen, 2012; Hou et al., 2020). The values of these metrics are higher, and the newly published articles are expected to have more potential to transform the intellectual base (Hou et al., 2020). Specifically, cluster linkage is a direct measure of intellectual potential and structural change (Chen, 2012). Therefore, we adopted cluster linkage as an indicator by which to recognize and predict the valuable ideas in newly published procrastination research. These top 20 articles with high transformative potential that were published during the period 2016-2020 were listed (see Supplementary Material for details). Research contents primarily consist of four dimensions.

#### Further Investigations Into Academic Procrastination

Although procrastination research has drawn mostly on samples of students, innovative research contents and methods have been emerging that enhance our understanding of academic procrastination. In the past five years, different language versions of scales have been measured and validated (Garzon Umerenkova and Gil-Flores, 2017a,b; Svartdal, 2017; Guilera et al., 2018), and novel research areas and contents have arisen, such as how gender difference influences academic procrastination, what are the effective means of intervention, and what are the associations among academic procrastination, person-environment fit, and academic achievement (Balkis and Duru, 2016; Garzon Umerenkova and Gil-Flores, 2017a,b; Goroshit, 2018). Interestingly, research has found that females perform academic procrastination less often and gain better academic achievements than males do (Balkis and Duru, 2017; Perdomo and Feliciano-Garcia, 2020).

In addition, academic procrastination is viewed as a fluid process. Considering the behavior holistically, three different aspects of task engagement have been discussed: initiation, completion, and pursuit. Vangsness and Young (2020) proposed the metaphors of “turtles” (steady workers), “task ninjas” (precrastinators), and “time wasters” (procrastinators) to elaborate vividly on task completion strategies when working toward deadlines. Individual differences and task characteristics can influence one's choices of a task-completion strategy. To understand the fluid and multifaceted phenomenon of procrastination, longitudinal research has been appearing. Wessel et al. (2019) observed behavioral delay longitudinally through tracking an undergraduate assignment over two weeks to reveal how passive and active procrastination each affected assignment completion.

#### Relationships Between Procrastination and Diverse Personality Traits

In addition to the relationship between procrastination and the five-factor model, other personality traits, such as temperament, character, emotional intelligence, impulsivity, and motivation, have been investigated in connection with procrastination. Because the five-factor model is not effective for distinguishing the earlier developing temperamental tendencies and the later developing character traits, Zohar et al. (2019) discussed how temperament and character influence procrastination in terms of active and passive procrastinators, and revealed that a dependable temperament profile and well-developed character predicted active procrastination.

Procrastination is commonly defined as a self-regulation failure that includes emotion and behavior. Emotional intelligence (EI) is an indicator with which to monitor one's feelings, thinking, and actions, and hot discussions about its relationship with procrastination have sprung up recently. Sheybani et al. (2017) elaborated on how the relationship between emotional intelligence and the five-factor model influence decisional procrastination on the basis of a students' sample. As a complement to the research above, Wypych et al. (2018) explored the roles of impulsivity, motivation, and emotion regulation in procrastination through path analysis. Motivation and impulsivity reflecting a lack of value, along with delay discounting and lack of perseverance, are predicators of procrastination, whereas emotion regulation, especially for suppression of procrastination, has only appeared to be significant in student and other low-age groups. How personality traits influence procrastination remains controversial, and further research is expected.

#### Procrastination in Different Life-Domains and Settings

Newly published research is paying more attention to procrastination in different sample groups across the entire life span. Not being limited to student samples, discussions about procrastination in groups such as teachers, educated adults, and workers have been emerging. With regard to different life domains, the self-oriented domains including health and leisure time, tend to procrastinate, whereas parenting is low in procrastination among highly educated adults. Although the achievement-oriented life domains of career, education, and finances are found with moderate frequency in conjunction with procrastination, these three domains together with health affect life the most (Hen and Goroshit, 2018). Similarly, Tibbett and Ferrari (2019) investigated the main regret domains facing cross-cultural samples, so as to determine which factors increased the likelihood of identifying oneself as a procrastinator. Their research found that forms of earning potential, such as education, finances, and career, led participants to more easily label themselves as procrastinators. Procrastination can lead to regret, and this research adopted reverse thinking to discuss the antecedents of procrastination.

In addition to academic procrastination, research about the behavior in diverse-context settings has begun to draw scholars' attention. Nauts et al. (2019) used a qualitative study to investigate why people delay their bedtime, and the study identified three forms of bedtime procrastination: deliberate procrastination, mindless procrastination, and strategic delay. Then, those researchers proposed coached interventions involving time management, priority-setting skills, and reminders according to the characteristics of the bedtime procrastination. Interestingly, novel forms of procrastination have been arising in the attention-shortage situations of the age of the internet, such as social media self-control failure (SMSCF). Du et al. (2019) found that habitual checking, ubiquity, and notifications were determinants for self-control failures due to social media use, and that finding provided insight into how to better use ICTs in a media-pervasive environment. Moreover, even beyond those life-related-context settings, procrastination in the workplace has been further explored. Hen (2018) emphasized the factor of professional role ambiguity underlying procrastination. Classification of procrastination context is important for the effectiveness of intervention and provides us with a better understanding of this multifaceted behavior.

#### Interventions to Procrastination

Overcoming procrastination is a necessary topic for discussion. Procrastination is prevalent and stable across situations, and it is commonly averse to one's performance and general well-being. Various types of interventions are used, such as time management, self-management, and cognitive behavioral therapy. To examine the effectiveness of those interventions, scholars have used longitudinal studies or field experimental designs to investigate these methods of intervention for procrastination. Rozental et al. (2017) examined the efficacy of internet-based cognitive behavior therapy (ICBT) to relieve procrastination, from the perspective of clinical trials. Through a one-year follow-up in a randomized controlled trial, researchers found that ICBT could be beneficial to relieve severe, chronic procrastination. Taking the temporal context into consideration, Visser et al. (2017) discussed a strengths-based approach — one element of the cognitive behavioral approach — that showed greater usefulness for students at an early stage of their studies than it did at later ages. Overall, research on the effectiveness of intervention for procrastination is relatively scarce.

## Discussion and Conclusion

### Discussion on Procrastination Research

**Figure 6:**
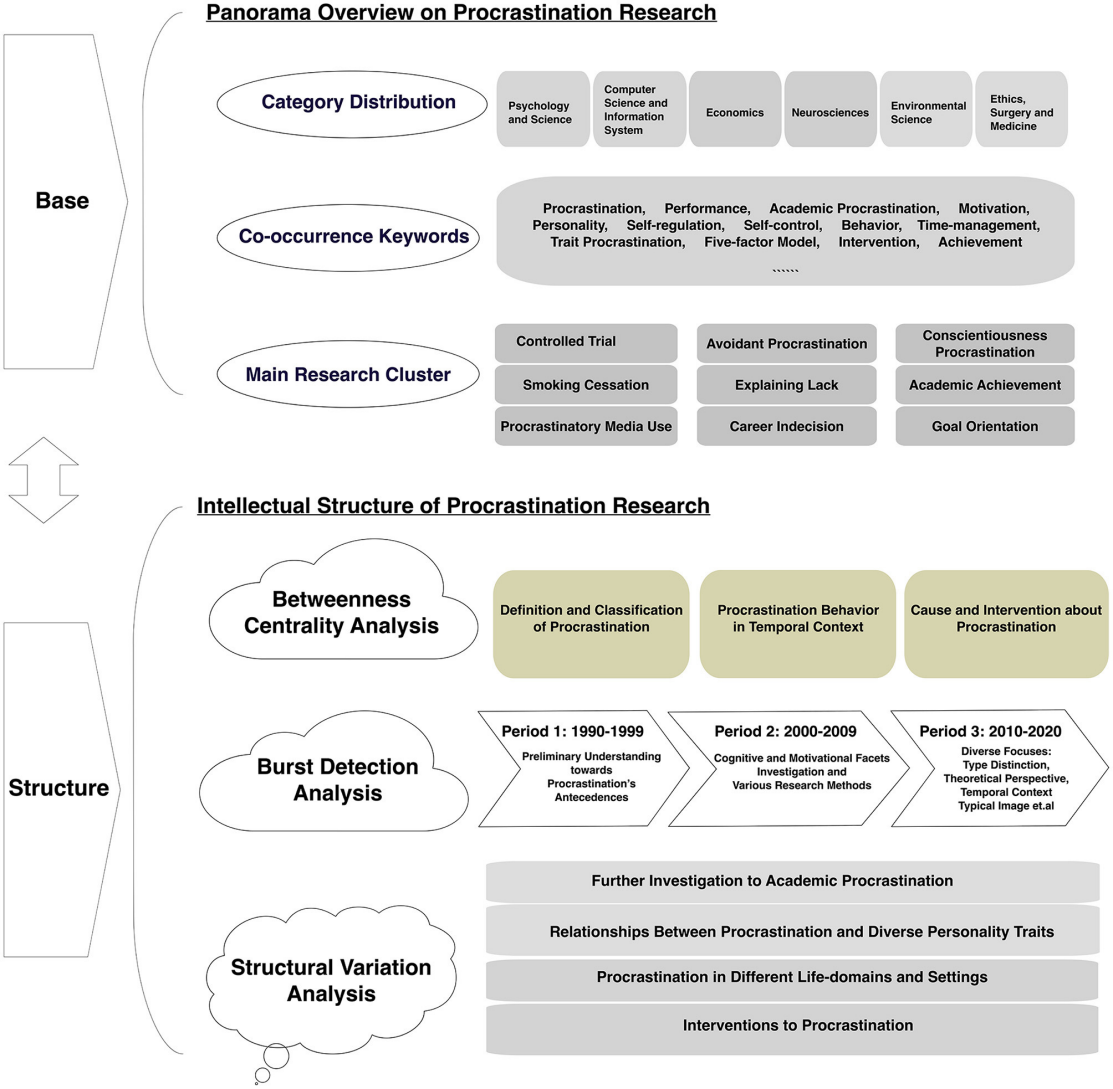
Bibliometric analysis and science map of the literature on procrastination.

This article provides a systematic bibliometric analysis of procrastination research over the past 30 years. The study identifies the category distribution, co-occurrence keywords, main research clusters, and intellectual structures, with the help of CiteSpace and VOS viewer. As is shown in Figure 6, the primary focuses for research themes have been on the definition and classification of procrastination, the relationships between procrastination and personality traits, the influences brought by procrastination, and how to better intervene in this complex phenomenon.

Those contents have built the bases for procrastination research, but determining how those bases are constructed is important to the development of future research. Therefore, this article primarily discusses three aspects of intellectual structure of procrastination research: betweenness centrality, burst detection, and structural variation analysis. From the betweenness centrality analysis, three research themes are identifiable and can be generally summarized as: definition and classification of procrastination, procrastination behavior in a temporal context, and causes and interventions for procrastination.

However, procrastination research themes have evolved significantly across the time period from 1990–2020. Through burst detection analysis, we are able to infer that research has paid extraordinary attention to diverse themes at different times. In the initial stage, research is mainly about the antecedents of procrastination from the perspectives of time-management, self-regulation failure, and the five-factor model, which pays more attention to the behavior itself, such as delays in time. Subsequently, further discussions have focused on how cognitive and motivational facets such as goal orientation, perceived self-efficacy, self-handicapping, as well as self-regulated learning strategies influence procrastination. In the most recent 10 years, research has paid significant attention to expanding diverse themes, such as theoretical perspectives, typical images of procrastinators, and procrastination behavior in diverse temporal contexts. Research about procrastination has been gaining more and more attention from scholars and practitioners.

To explore newly published articles and their transformative potential, we conduct structural variation analysis. Beyond traditional research involving academic procrastination, emerging research themes consist of diverse research settings across life-domains, such as bedtime procrastination, social media self-control failure, procrastination in the workplace, and procrastination comparisons between self-oriented and achievement-oriented domains. Furthermore, novel interventions from the perspective of clinical and cognitive orientations to procrastination have been emerging in response to further investigation of procrastination's antecedents, such as internet-based cognitive behavior therapy (ICBT) and the strengths-based approach.

### Conclusions and Limitations

**Figure 7:**
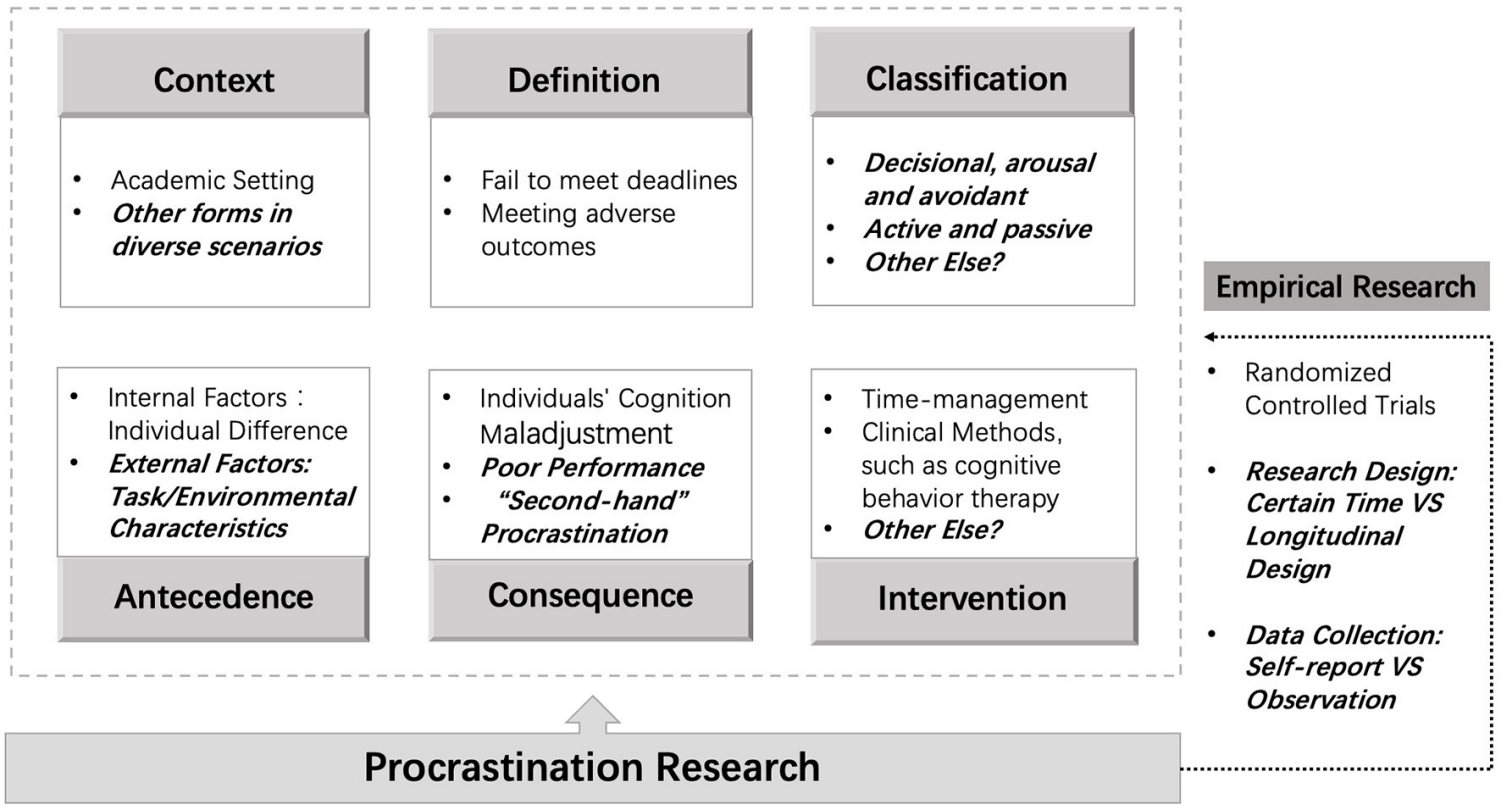
Brief conclusions on procrastination research.

In summary, research on procrastination has gained increasing attention during 1990 to 2020. Specifically in Figure 7, research themes have involved in the definition, classification, antecedents, consequences, interventions, and diverse forms of procrastination across different life-domains and contexts. Furthermore, empirical research has been conducted to understand this complex and multifaceted behavior, including how best to design controlled trial experiments, how to collect and analyze the data, and so on.

From the perspective of knowledge development, related research about procrastination has experienced tremendous expansion in the last 30 years. There are three notable features to describe the evolutionary process.

First, research focuses are moving from broader topics to more specific issues. Prior research mostly explored the definition and antecedents of procrastination, as well as the relationship between personality traits and procrastination. Besides, earlier procrastination research almost drew on students' setting. Based on previous research above, innovative research starts to shed light on procrastination in situation-specific domains, such as work procrastination, bedtime procrastination, as well as the interaction between problematic new media use and procrastination (Hen, 2018; Nauts et al., 2019; Przepiorka et al., 2021). With the evolvement of research aimed at distinct contexts, more details and core contents about procrastination have been elaborated. For example, procrastination in workplace may have association with professional role ambiguity, abusive supervision, workplace ostracism and task characteristics (Hen, 2018; He et al., 2021; Levin and Lipshits-Braziler, 2021). In particular, owing to the use of information and communication technology (ICTs), there currently are ample temptations to distract our attention, and those distractions can exacerbate the severity of procrastination (Du et al., 2019; Hong et al., 2021). Therefore, how to identify those different forms of procrastination, and then to reduce their adverse outcomes, will be important to discuss.

Second, antecedents and consequences of procrastination are further explored over time. On one hand, how procrastination occurs arises hot discussions from diverse dimensions including time management, personality traits, contextual characteristics, motivational and cognitive factors successively. Interestingly, investigations about neural evidences under procrastination have been emerging, such as the underlying mechanism of hippocampal-striatal and amygdala-insula to procrastination (Zhang et al., 2021). Those antecedents can be divided into internal factors and external factors. Internal factors including character traits and cognitive maladjustments have been elucidated fully, but scant discussion has occurred about how external factors, such as task characteristics, peers' situations, and environmental conditions, influence procrastination (Harris and Sutton, 1983; He et al., 2021). On the other hand, high prevalence of procrastination necessitates the importance to identify the negative consequences including direct and indirect. Prior research paid more attention to direct consequences, such as low performance, poor productivity, stress and illness, but the indirect consequences that can be brought about by procrastination remain to be unclear. For example, “second-hand” procrastination vividly describes the “spillover effect” of procrastination, which is exemplified by another employee often working harder in order to compensate for the lost productivity of a procrastinating coworker (Pychyl and Flett, 2012). Although such phenomena are common, adverse outcomes are less well investigated. Combining the contexts and groups involved, targeted discussions about the external antecedents and indirect consequences of procrastination are expected.

Third, empirical research toward procrastination emphasizes more on validity. When it comes to previous research, longitudinal studies are often of small numbers. However, procrastination is dynamic, so when most studies focus on procrastination of students' sample during just one semester or several weeks, can limit the overall viewpoints about procrastination and the effectiveness of conclusions. With the development of research, more and more longitudinal explorations are springing up to discuss long-term effects of procrastination through behavioral observation studies and so on. Besides, how to design the research and collect data evolves gradually. Self-reported was the dominant method to collect data in prior research, and measurements of procrastination usually depended on different scales. However, self-reported data are often distorted by personal processes and may not reflect the actual situation, even to overestimate the level of procrastination (Kim and Seo, 2015; Goroshit, 2018). Hence, innovative studies start to conduct field experimental designs to get observed information through randomized controlled trials. For the following research, how to combine self-reported data and observed data organically should be investigated and refined.

This bibliometric analysis to procrastination is expected to provide overall perspective for future research. However, certain limitations merit mentioning here. Owing to the limited number of pages allowed, it is difficult to clarify the related articles in detail, so discussion tends to be heuristic. Furthermore, the data for this research comes from the Web of Science database, and applying the same strategy to a different database might have yielded different results. In the future, we will conduct a systematic analysis using diverse databases to detect pivotal articles on procrastination research.

## Data Availability Statement

The original contributions presented in the study are included in the article/Supplementary Material, further inquiries can be directed to the corresponding author/s.

## Author Contributions

BY proposed the research question and conducted the research design. XZ analyzed the data and wrote primary manuscript. On the base of that work mentioned above, two authors discussed and adjusted the final manuscript together.

## Conflict of Interest

The authors declare that the research was conducted in the absence of any commercial or financial relationships that could be construed as a potential conflict of interest.

## Publisher's Note

All claims expressed in this article are solely those of the authors and do not necessarily represent those of their affiliated organizations, or those of the publisher, the editors and the reviewers. Any product that may be evaluated in this article, or claim that may be made by its manufacturer, is not guaranteed or endorsed by the publisher.
