# Supplementary material for: What Research Has Been Conducted on Procrastination? Evidence From a Systematical Bibliometric Analysis
Source: Front Psychol. 2022 Feb 2;13:809044. doi: 10.3389/fpsyg.2022.809044 (PMC8847795; doi:10.3389/fpsyg.2022.809044)
Supplement: Supplementary file 1 [file Table_1.docx]

**Supplementary Material**

**Table1 Top 10 Articles with High Betweenness Centrality**

| **Centrality** | **References** |
| --- | --- |
| 44 | Sirois, F., & Pychyl, T. (2013). Procrastination and the Priority of Short-Term Mood Regulation: Consequences for Future Self. Social and Personality Psychology Compass, 7(2), 115-127. |
| 41 | Johnson, J. L., & Bloom, A. M. (1995). An analysis of the contribution of the five factors of personality to variance in academic procrastination. Personality and Individual Differences, 18(1), 127-133. |
| 37 | Ferrari, J. R., Diaz-Morales, J. F., O'Callaghan, J., Diaz, K., & Argumedo, D. (2007). Frequent behavioral delay tendencies by adults - International prevalence rates of chronic procrastination. Journal of Cross-Cultural Psychology, 38(4), 458-464. |
| 36 | Corkin, D. M., Yu, S. L., & Lindt, S. F. (2011). Comparing active delay and procrastination from a self-regulated learning perspective. Learning and Individual Differences, 21(5), 602-606. |
| 35 | Glick, D. M., & Orsillo, S. M. (2015). An investigation of the efficacy of acceptance-based behavioral therapy for academic procrastination. Journal of Experimental Psychology: General, 144(2), 400–409. |
| 34 | Steel, P. (2010). Arousal, avoidant and decisional procrastinators: Do they exist? Personality and Individual Differences, 48(8), 926-934. |
| 34 | Diaz-Morales, J. F., Ferrari, J. R., Diaz, K., & Argumedo, D. (2006). Factorial structure of three procrastination scales with a Spanish adult population. European Journal of Psychological Assessment, 22(2), 132-137. |
| 32 | Tice, D. M., & Baumeister, R. F. (1997). Longitudinal study of procrastination, performance, stress, and health: The costs and benefits of dawdling. Psychological Science, 8(6), 454-458. |
| 32 | Balkis, M. (2013). Academic procrastination, academic life satisfaction and academic achievement: The mediation role of rational beliefs about studying. Journal of Cognitive and Behavioral Psychotherapies, 13, 57-74. |
| 31 | Gustavson, D. E., Miyake, A., Hewitt, J. K., & Friedman, N. P. (2014). Genetic Relations Among Procrastination, Impulsivity, and Goal-Management Ability: Implications for the Evolutionary Origin of Procrastination. Psychological Science, 25(6), 1178-1188. |

**Table2 Top 20 References with the Strongest Citation Bursts**

| **Period** | **Bursts** | **References** |
| --- | --- | --- |
| I | 12.36 | Lay, C. (1988). The relation of procrastination and optimism to judgments of time to complete an essay and anticipation of setbacks. Journal of Social Behavior & Personality, 3, 201-214. |
|  | 11.84 | Ferrari, J. R. (1991). Compulsive procrastination: some self-reported characteristics. Psychological Reports, 68(2), 455-458. |
|  | 10.79 | Ferrari, J. R. (1992). Psychometric validation of two Procrastination inventories for adults: Arousal and avoidance measures. Journal of Psychopathology and Behavioral Assessment, 14(2), 97-110. |
|  | 31.36 | Ferrari, J.R., Johnson, J.L., & McCown, W.G. (1995). Procrastination and task avoidance: Theory, research, and treatment. Contemporary Psychology, 41 (7): 698-699. |
|  | 13.05 | Schouwenburg, HC & Lay CH. (1995). Trait procrastination and the Big-five factors of personality. Personality and Individual Differences, 18(4): 481-490. |
|  | 19.05 | Tice, D. M., & Baumeister, R. F. (1997). Longitudinal Study of Procrastination, Performance, Stress, and Health: The Costs and Benefits of Dawdling. Psychological Science, 8(6): 454–458. |
| II | 11.15 | Ariely, D., & Wertenbroch, K. (2002). Procrastination, deadlines, and performance: Self-control by precommitment. Psychological Science, 13(3), 219-224. |
|  | 11.51 | Wolters, C. A. (2003). Understanding procrastination from a self-regulated learning perspective. Journal of Educational Psychology, 95(1), 179-187. |
|  | 13.46 | van Eerde, W. (2003). A meta-analytically derived nomological network of procrastination. Personality and Individual Differences, 35(6), 1401-1418. |
|  | 18.92 | Chu, A. H. C., & Choi, J. N. (2005). Rethinking procrastination: Positive effects of "active" procrastination behavior on attitudes and performance. Journal of Social Psychology, 145(3), 245-264. |
|  | 69.02 | Steel P. (2007). The nature of procrastination: a meta-analytic and theoretical review of quintessential self-regulatory failure. *Psychol Bull*, 133(1):65-94. |
|  | 18.44 | Howell, A. J., & Watson, D. C. (2007). Procrastination: Associations with Achievement Goal Orientation and Learning Strategies. Personality and Individual Differences, 43(1), 167-178. |
|  | 20.63 | Schraw, G., Wadkins, T., & Olafson, L. (2007). Doing the things we do: A grounded theory of academic procrastination. Journal of Educational Psychology, 99(1), 12–25. |
|  | 19.96 | Klassen RM, Krawchuk LL, & Rajani S. (2008). Academic procrastination of undergraduates: Low self-efficacy to self-regulate predicts higher levels of procrastination. Contemporary Educational Psychology, 33(4): 915-931. |
| III | 20.18 | Steel P. (2010). Arousal, avoidant and decisional procrastinators: Do they exist? Personality and Individual Differences, 48 (8): 926-934. |
|  | 11.84 | Sirois, F., & Pychyl, T. (2013). Procrastination and the Priority of Short-Term Mood Regulation: Consequences for Future Self. Social and Personality Psychology Compass, 7(2), 115-127. |
|  | 14.57 | Klingsieck, K. B. (2013). Procrastination: When Good Things Don't Come to Those Who Wait. European Psychologist, 18(1), 24-34. |
|  | 11.11  20.07 | Steel, P., & Ferrari, J. (2013). Sex, Education and Procrastination: An Epidemiological Study of Procrastinators' Characteristics from a Global Sample. *European Journal of Personality, 27*(1), 51-58.  Kim KR & Seo EH. (2015). The relationship between procrastination and academic performance: A meta-analysis. Personality and Individual Differences, 82: 26-33. |
|  | 15.81 | Piers Steel & Katrin B Klingsieck (2016) Academic Procrastination: Psychological Antecedents Revisited, Australian Psychologist, 51:1, 36-46. |

**Table3 Top 20 Articles with High Potential Transformation During 2016-2020**

| **Year** | **Cluster Linkage** | **Reference** |
| --- | --- | --- |
| 2019 | 8.5396 | Wessel, J., Bradley, G. L., & Hood, M. (2019). Comparing effects of active and passive procrastination: A field study of behavioral delay. Personality and Individual Differences, 139, 152-157. |
| 2017 | 7.6201 | Sheybani, F., Gharraee, B., Bakhshizadeh, M., & Tamanaeefar, S. (2017). Decisional procrastination: prevalence among students and relationship with emotional intelligence and big five-factor model of personality. International Journal of Life Science and Pharma Research, 7(2), L26-L32. |
| 2018 | 7.5839 | Hen, M. (2018). Causes for procrastination in a unique educational workplace. Journal of Prevention & Intervention in the Community, 46(3), 215-227. |
| 2018 | 7.2987 | Guilera, G., Barrios, M., Penelo, E., Morin, C., Steel, P., & Gomez-Benito, J. (2018). Validation of the Spanish version of the Irrational Procrastination Scale (IPS). PLoS One, 13(1). |
| 2020 | 6.7678 | Perdomo, A. S., & Feliciano-Garcia, L. (2020). The influence of active procrastination: a profile on Educational Sciences students' academic achievement. Bordon-Revista De Pedagogia, 72(3), 157-170. |
| 2018 | 6.7314 | Goroshit, M. (2018). Academic procrastination and academic performance: An initial basis for intervention. Journal of Prevention & Intervention in the Community, 46(2), 131-142. |
| 2019 | 6.667 | Nauts, S., Kamphorst, B. A., Stut, W., De Ridder, D. T. D., & Anderson, J. H. (2019). The Explanations People Give for Going to Bed Late: A Qualitative Study of the Varieties of Bedtime Procrastination. Behavioral Sleep Medicine, 17(6), 753-762. |
| 2017 | 6.3547 | Garzon-Umerenkova, A., & Gil-Flores, J. (2017). Academic procrastination in non-traditional college students. Electronic Journal of Research in Educational Psychology, 15(3), 510-531. |
| 2017 | 5.7143 | Svartdal, F. (2017). Measuring procrastination: Psychometric properties of the Norwegian versions of the Irrational Procrastination Scale (IPS) and the Pure Procrastination Scale (PPS). Scandinavian Journal of Educational Research, 61(1), 18-30. |
| 2017 | 5.4469 | Rozental, A., Forsell, E., Svensson, A., Andersson, G., & Carlbring, P. (2017). Overcoming procrastination: one-year follow-up and predictors of change in a randomized controlled trial of Internet-based cognitive behavior therapy. Cognitive Behaviour Therapy, 46(3), 177-195. |
| 2019 | 5.4378 | Ferrari, J., & Tibbett, T. (2019). Return to the origin: what creates a procrastination identity? Current Issues in Personality Psychology, 6. |
| 2018 | 5.1699 | Wypych, M., Matuszewski, J., & Dragan, W. L. (2018). Roles of Impulsivity, Motivation, and Emotion Regulation in Procrastination - Path Analysis and Comparison Between Students and Non-students. Frontiers in psychology, 9, 10. |
| 2018 | 5.1324 | Hen, M., & Goroshit, M. (2018). General and Life-Domain Procrastination in Highly Educated Adults in Israel. Frontiers in psychology, 9. |
| 2020 | 5.0806 | Vangsness, L., & Young, M. E. (2020). Turtle, Task Ninja, or Time Waster? Who Cares? Traditional Task-Completion Strategies Are Overrated. *Psychological Science, 31*(3), 306-315. |
| 2017 | 5.0443 | Balkis, M., & Duru, E. (2017). Gender Differences in the Relationship between Academic Procrastination, Satisfaction with Academic Life and Academic Performance. Electronic Journal of Research in Educational Psychology, 15(1), 105-125. |
| 2017 | 5.0132 | Garzon Umerenkova, A., & Gil-Flores, J. (2017). Psychometric Properties of the Spanish Version of the Test Procrastination Assessment Scale-Students (PASS). Revista Iberoamericana De Diagnostico Y Evaluacion-E Avaliacao Psicologica, 1(43), 149-163. |
| 2019 | 5 | Du, J., Kerkhof, P., & van Koningsbruggen, G. M. (2019). Predictors of Social Media Self-Control Failure: Immediate Gratifications, Habitual Checking, Ubiquity, and Notifications. Cyberpsychology Behavior and Social Networking, 22(7), 477-485. |
| 2016 | 5 | Balkis, M., & Duru, E. (2016). The Analysis of Relationships among Person-Environment Fit, Academic Satisfaction, Procrastination and Academic Achievement. Pamukkale Universitesi Egitim Fakultesi Dergisi-Pamukkale University Journal of Education (39), 119-129. |
| 2019 | 4.9471 | Zohar, A. H., Shimone, L. P., & Hen, M. (2019). Active and passive procrastination in terms of temperament and character. Peerj, 7, 16. |
| 2017 | 4.8417 | Visser, L., Schoonenboom, J., & Korthagen, F. A. J. (2017). A Field Experimental Design of a Strengths-Based Training to Overcome Academic Procrastination: Short- and Long-Term Effect. Frontiers in psychology, 8, 12. |
